# Meetings of Societies

**Published:** 1946

**Authors:** 


### Meetings of Societies

### Bristol Medico-Chirurgical Society

The sixth meeting held in April was the occasion of a lively addres on "**Some Aspects of Industrial Medicine in Great Britain at War,**" by Mr. Donald Hunter, M.D., M.B., B.S., F.R.C.S. The speaker covered an interesting series of his experiences in this field and leavened his address with much humour.

The seventh meeting, held in May, was devoted to short papers. Three were given :—

"**Symptomless Hyperpiesia in Dockers,**" by Dr. J. N. Naish, M.D. M.R.C.P.

"**Transitory Generalized Paralysis,**" by Dr. G. de M. Rudolf, M.R.C.P., D.P.M.

"**Treatment of Perthe's Disease by Traction,**" by Mr. A. L., Eyre-Brook, F.R.C.S.

